# Changes in incretin hormone concentrations after pancreaticoduodenectomy: a systematic review and exploratory meta-analysis

**DOI:** 10.3389/fendo.2026.1845925

**Published:** 2026-06-03

**Authors:** Oliwia Grząsiak-Kraj, Tomasz Kraj, Krzysztof Poznański, Aneta Szmiel, Janusz Strzelczyk

**Affiliations:** 1 Department of General and Transplant Surgery, Medical University of Lodz, Lodz, Poland; 2 Department of Vascular Surgery and Angiology, Independent Public Healthcare Institution of the Ministry of the Interior and Administration in Lodz, Lodz, Poland; 3 Institute of Medical Expertises in Lodz, Lodz, Poland

**Keywords:** enteroendocrine, enteroinsular axis, gastric emptying, GIP, GLP-1, glucose metabolism, incretin effect, incretins

## Abstract

**Background:**

Pancreaticoduodenectomy (PD) removes the duodenum and proximal jejunum, alters nutrient transit, and may modify secretion of incretin hormones involved in glucose homeostasis.

**Objective:**

To systematically review clinical evidence on postoperative changes in glucagon-like peptide-1 (GLP-1) and glucose-dependent insulinotropic polypeptide (GIP) after PD, together with co-reported hormonal and metabolic outcomes, and to perform a strictly exploratory meta-analysis where sufficiently comparable numerical data were available.

**Methods:**

Following PRISMA 2020, we reviewed human studies identified in PubMed, Embase, and Scopus from 1 January 2000 to 27 March 2026, with screening and full-text selection performed in Rayyan. Eligible studies reported GLP-1 and/or GIP after PD, after different PD reconstructions, or during post-PD nutrient-delivery interventions. Because outcome reporting was heterogeneous, quantitative pooling was restricted to exploratory random-effects syntheses of two clinically comparable post-PD reconstruction studies. Because only two studies contributed to each pooled comparison, estimates of effect size and heterogeneity were considered highly fragile and were not interpreted as confirmatory.

**Results:**

Six studies involving 134 participants met the eligibility criteria. Postprandial GLP-1 generally increased after PD or was higher in settings associated with faster gastric emptying or greater distal nutrient exposure. By contrast, GIP was reported in fewer studies than GLP-1 and showed attenuation or no consistent enhancement. Co-reported glucose, insulin, C-peptide, glucagon, PYY, and gastric-emptying measures indicated that endocrine adaptation after PD is heterogeneous and not uniformly beneficial despite the recurrent GLP-1 signal. Exploratory meta-analysis of Whipple versus pylorus-preserving PD comparisons suggested a direction favoring Whipple for early GLP-1 exposure, although confidence intervals were wide and the two-study pooled estimates were statistically fragile. Given the small number of heterogeneous studies, quantitative findings should be interpreted as exploratory.

**Conclusions:**

Available clinical evidence supports a reproducible increase in postprandial GLP-1 signaling after PD, whereas evidence for GIP is weaker, based on fewer studies, and remains sparse and inconsistent. The meta-analytic component should be regarded as exploratory and hypothesis-generating only. Larger prospective studies with standardized stimulation protocols and detailed assay reporting are needed.

**Systematic review registration:**

https://www.crd.york.ac.uk/prospero/, identifier 1345848.

## 1 Introduction

Pancreaticoduodenectomy is one of the most profound gastrointestinal reconstructions performed in abdominal surgery (1–3). In addition to reducing pancreatic tissue mass, PD removes the duodenum, bypasses the proximal jejunum, and changes the tempo and topology of nutrient delivery to more distal bowel segments (1–3). These changes are mechanistically relevant to the enteroinsular axis, particularly to GLP-1 and GIP secretion, because L-cell stimulation depends strongly on distal nutrient exposure while K-cell signaling is concentrated more proximally (1–3). Over the last two decades, several small clinical studies have reported that postprandial GLP-1 concentrations rise after PD or are higher after reconstructions that accelerate gastric emptying and enhance distal nutrient exposure (4–25). However, the direction and consistency of accompanying changes in GIP, insulin, glucagon, glucose excursions, and insulin sensitivity have been less clear (4–25). The evidence base is fragmented across perioperative cohorts, reconstruction comparisons, and feeding-route experiments, with substantial variation in postoperative timing, hormonal assays, and stimulation protocols (4–25). A broader 2020 systematic review of metabolic dysfunction after PD and duodenum-preserving total pancreatic head resection summarized endocrine and exocrine outcomes and reported increased GLP-1 and glucagon responses after PD, but it was not focused on incretin physiology, pooled diverse endocrine outcomes together, and predated several mechanistic studies that sharpened interest in the incretin axis after PD (1). Our review therefore aimed to provide a focused synthesis of GLP-1 and GIP after PD, while also integrating co-reported changes in glucose, insulin, glucagon, Cpeptide, PYY, neurotensin, motilin, and gastric emptying (1). We hypothesized that the dominant post-PD signal would be an increase in GLP-1, with the largest responses in settings that maximize rapid distal intestinal exposure to nutrients, and that evidence for GIP would be sparser and more heterogeneous.

## 2 Methods

This systematic review was conducted in accordance with the PRISMA 2020 statement and prepared in the format required for Frontiers in Endocrinology (26–29). The review protocol was prospectively registered in PROSPERO (registration number: 1345848) (26–29). The protocol was registered prospectively before completion of study selection and data synthesis (26–29).

Eligibility criteria were structured according to PICOS. Population: humans undergoing PD/Whipple/PPPD. Intervention or exposure: PD itself, reconstruction variant after PD, or post-PD nutrient delivery route. Comparator: preoperative state, alternative reconstruction, alternative feeding route, or post-PD subgroups. Outcomes: GLP-1 or GIP concentrations measured fasting and/or after a standardized oral or enteral stimulus; co-reported insulin, glucagon, glucose, C-peptide, PYY, neurotensin, motilin, gastric emptying, or insulin sensitivity were also extracted. Study designs: prospective or observational clinical studies with at least 10 participants in the PD-relevant analytic group. Reviews, case reports, non-human studies, studies without incretin measurement, and studies dominated by non-PD populations were excluded. Search strategies documented in the study workflow were applied in PubMed, Embase, and Scopus from 1 January 2000 to 27 March 2026, with restriction to the English language (27, 28). The search strategy combined controlled vocabulary (e.g., MeSH and Emtree terms) with free-text keywords and was iteratively developed and refined by the authors (27, 28). The final strategy was independently reviewed by a second investigator (T.K.) for completeness, accuracy of controlled vocabulary, and appropriate use of Boolean operators, in line with PRISMA-S recommendations (27, 28). Rayyan was used for duplicate removal, title/abstract screening, and full-text eligibility assessment, with screening performed independently by two reviewers and disagreements resolved by consensus (27, 28). A native Rayyan customization-log CSV export was additionally used to verify final full-text decisions and record-level exclusion reasons (27, 28). The Rayyan workflow overview indicated 226 imported references, 103 duplicates deleted, 123 records screened, 36 full texts assessed, and 6 studies included (27, 28).

From each eligible study, we extracted country, design, sample size, surgical technique, comparator structure, postoperative timing, stimulation test, hormone assay, and quantitative hormonal and metabolic outcomes. Where only medians and ranges/interquartile ranges were reported, these summary statistics were retained for narrative synthesis and transformed for exploratory meta-analysis only. Figure-based estimates present in the extraction workbook were treated as digitized approximations and were not privileged over tabulated data when both were available. Risk of bias was assessed using a design-adapted framework informed by ROBINS-I domains, applying a pragmatic domain-based approach tailored to before-after studies, cross-sectional comparative studies, and observational postoperative cohorts (29). A full ROBINS-I assessment was not feasible due to incomplete reporting and heterogeneity of study designs; therefore, a domain-based assessment was applied (29). Core domains included selection bias, validity of hormonal assay measurement, completeness of follow-up and sampling, confounding, and appropriateness of statistical reporting (30–35). Overall certainty of evidence for the main GLP-1 signal was appraised qualitatively using GRADE principles (30–35). Because outcome reporting was heterogeneous, formal pooling was restricted *a priori* to outcomes with at least two clinically comparable studies reporting post-PD Whipple versus PPPD contrasts in the same unit (30–35). We identified two such primary outcomes—GLP-1 peak and GLP-1 AUC30—from Harmuth et al. and Steiner et al. In addition, exploratory secondary pooling was performed for insulin AUC30 and glucose AUC30, as these outcomes were also reported in both reconstruction-comparison studies and were considered sufficiently comparable for hypothesis-generating quantitative synthesis. These secondary analyses were intended to provide supportive metabolic context for the primary GLP-1 findings and were interpreted with the same caution as the pooled GLP-1 estimates. To permit synthesis, medians with ranges or IQRs were converted to approximate means and SDs using established methods for summary-statistic transformation (30–35). Random-effects pooling was then performed using Hedges g standardized mean differences (30–35). Statistical heterogeneity was assessed using the I² statistic; however, because each pooled comparison included only two studies, I² and effect-size estimates were considered highly fragile and were not treated as stable estimates of heterogeneity or comparative effect (30–35). All pooled results should therefore be interpreted as strictly exploratory because they rely on transformed nonparametric summaries from small observational datasets and two-study random-effects models (30–35). Before effect-size calculation, outcome definitions and reporting units were checked for consistency (30–35). Where necessary, hormone concentrations were aligned to a common unit before deriving standardized mean differences; for the two pooled GLP-1 reconstruction comparisons, the reported metrics were judged sufficiently comparable after transformation of nonparametric summaries to approximate means and SDs (30–35). Given the very limited number of studies available for quantitative synthesis, no formal assessment of publication bias (e.g., funnel plot or statistical tests) was performed, in line with methodological recommendations for small meta-analyses (30–35).

## 3 Results

### 3.1 Study selection and characteristics

**Figure 1:**
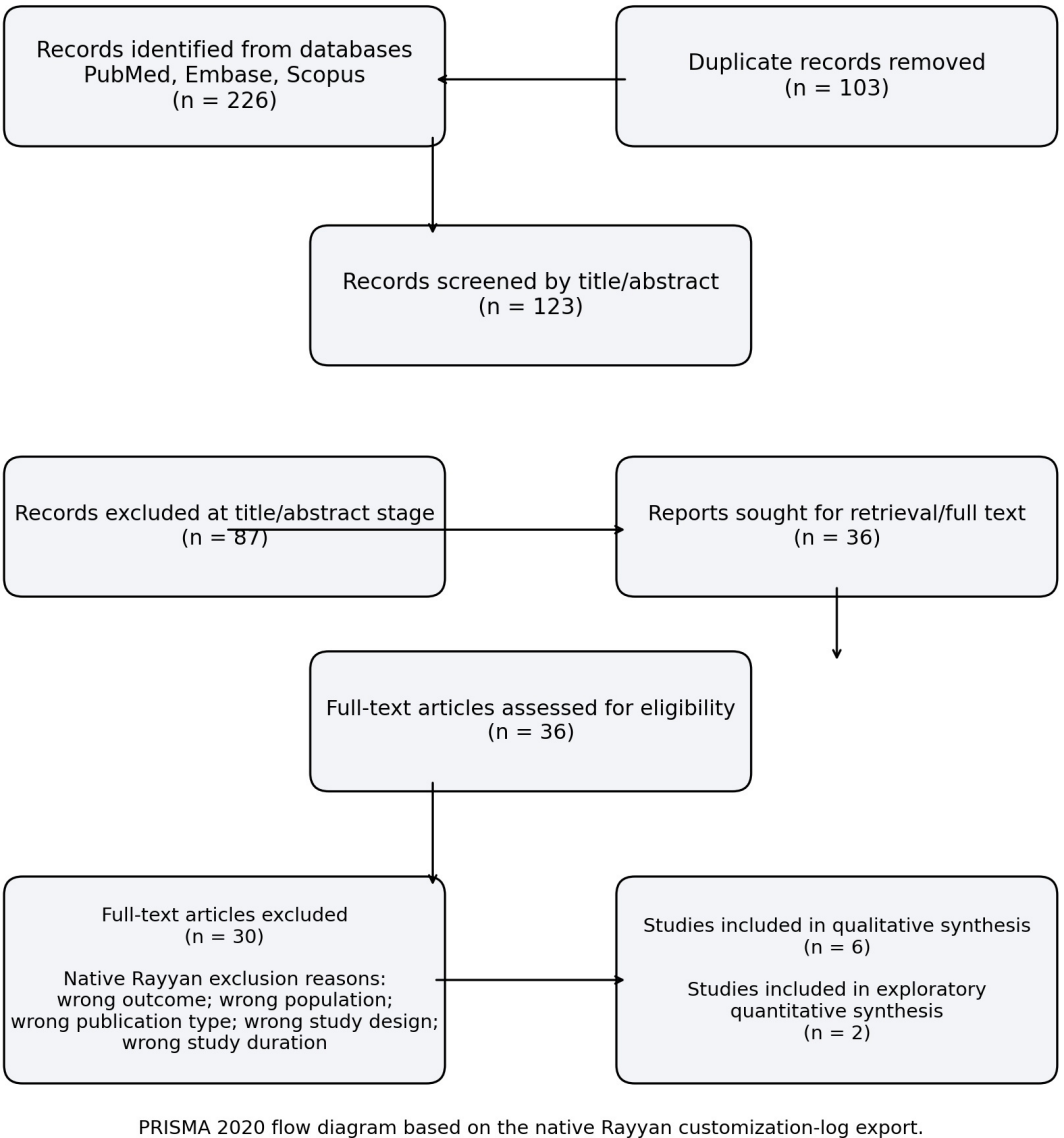
PRISMA 2020 flow diagram based on the native Rayyan customization-log export.

Six studies with 134 participants were included: two before-after PD cohorts (Muscogiuri 2013 and Ohtsuka 2009), one early postoperative cohort stratified by delayed gastric emptying (Strömmer 2005), two post-PD reconstruction comparisons (Harmuth 2014 and Steiner 2019), and one within patient feeding-route crossover experiment early after PD (Wu 2015) (4–9). Four studies measured GLP-1 alone or primarily GLP-1, whereas two also provided GIP data (4–9). Sample sizes ranged from 10 to 31 participants (4–9). No additional eligible studies were identified through reference screening or other sources (4–9). The study selection process is shown in **Figure 1**, and the characteristics of the included studies are summarized in **Table 1**.

**Table 1:** Characteristics of included studies.

Study	Design/comparison	n	Stimulus	Hormones	Main incretin finding
Muscogiuri 2013	Before-after PPPD	10	Mixed meal	GLP-1, GIP, glucagon, insulin, C-peptide, glucose	GLP-1 increased; GIP decreased after PPPD
Ohtsuka 2009	Before-after PD	17	75-g OGTT	GLP-1, insulin, glucose	Postoperative GLP-1 higher; glucose and insulin lower at 1 month
Strömmer 2005	DGE vs non-DGE after PD	31	Liquid meal + paracetamol	GLP-1, motilin, PYY, neurotensin	GLP-1 similar between groups; PYY and neurotensin lower with DGE
Harmuth 2014	Whipple vs PPPD after PD	26	75-g OGTT + paracetamol	GLP-1, insulin, glucose	Whipple associated with higher GLP-1 peak and AUC
Wu 2015	Proximal vs distal feeding after PD	20	Enteral meal test	GLP-1, GIP, insulin, C-peptide, glucose	Distal feeding increased GLP-1, insulin, and C-peptide AUCs; lowered glucose AUC
Steiner 2019	Whipple vs PPPD after PD	30	Mixed meal + paracetamol	GLP-1, insulin, glucose	Whipple and faster emptying associated with greater GLP-1 release

### 3.2 Qualitative synthesis of hormonal and metabolic outcomes

In the dedicated before-after incretin study, Muscogiuri et al. showed that PPPD increased GLP-1 and glucagon responses while reducing GIP, insulin, and C-peptide, with fasting and postprandial glucose increasing after surgery (4). This pattern suggests that enhanced GLP-1 secretion does not fully compensate for the abrupt reduction in pancreatic insulin secretory capacity in the early postoperative state (4). Ohtsuka et al. likewise reported higher postoperative GLP-1 concentrations 1 month after PD, but in contrast to Muscogiuri et al. the overall glucose profile improved transiently, with lower glucose and insulin concentrations and normalization of insulin resistance (5). Taken together, these two before-after studies support a reproducible GLP-1 rise after PD, while showing that downstream glucose handling may still depend on preoperative diabetic phenotype, postoperative timing, and remnant pancreatic function (5). Two reconstruction-comparison studies converged on the same mechanistic signal: distal gastrectomy/standard Whipple was associated with faster gastric emptying and higher postprandial GLP-1 release than PPPD (7, 9, 19–23, 36–40). Harmuth et al. reported higher GLP-1 peak, AUC30, and AUC180 after Whipple, whereas Steiner et al. found similar differences using a mixed-meal test and further linked higher GLP-1 exposure to better insulin sensitivity and lower HbA1c (7, 9, 19–23, 38–40). The feeding-route experiment by Wu et al. provided an elegant within-patient confirmation of the hindgut/distal exposure concept (8). Delivering the same nutrients more distally increased GLP-1, insulin, and C-peptide exposure while lowering glucose AUC (8). In the same study, GIP changed little, reinforcing the impression that GIP is not the dominant mediator of the post-PD metabolic signal (8). Strömmer et al. studied patients early after PD and found that GLP-1 responses were similar in patients with and without delayed gastric emptying, whereas PYY and neurotensin responses were reduced in those with delayed emptying (6). These data suggest that not all distal gut hormones behave identically in the early postoperative phase and that delayed nutrient progression may blunt selected distal peptide signals more than GLP-1 (6). The direction of hormonal and metabolic effects across the included studies is summarized in **Table 2**.

**Table 2:** Summary of the direction of hormonal and metabolic effects.

Outcome domain	Direction of effect across the included studies	Interpretation
GLP-1	Consistently increased after PD or under conditions of greater distal nutrient exposure	Most reproducible hormonal signal across the evidence base
GIP	Decreased after PPPD in 1 before-after study; largely unchanged with distal feeding in 1 crossover study	Sparse and inconsistent evidence
Insulin/C-peptide	Often lower after PD overall, but increased acutely when nutrients were delivered more distally	Depends on residual beta-cell capacity and test context
Glucose	Mixed overall; lower exposure after distal feeding and in one early postoperative before-after study	Not uniformly improved despite GLP-1 rise
Glucagon	Increased after PPPD in the dedicated before-after incretin study	May reflect reduced insulin restraint and altered gut glucagon biology
PYY/neurotensin/motilin	Reduced PYY and neurotensin with delayed gastric emptying; motilin similar	Suggests selective disruption of distal gut signaling in DGE

### 3.3 Exploratory quantitative synthesis

**Figure 2:**
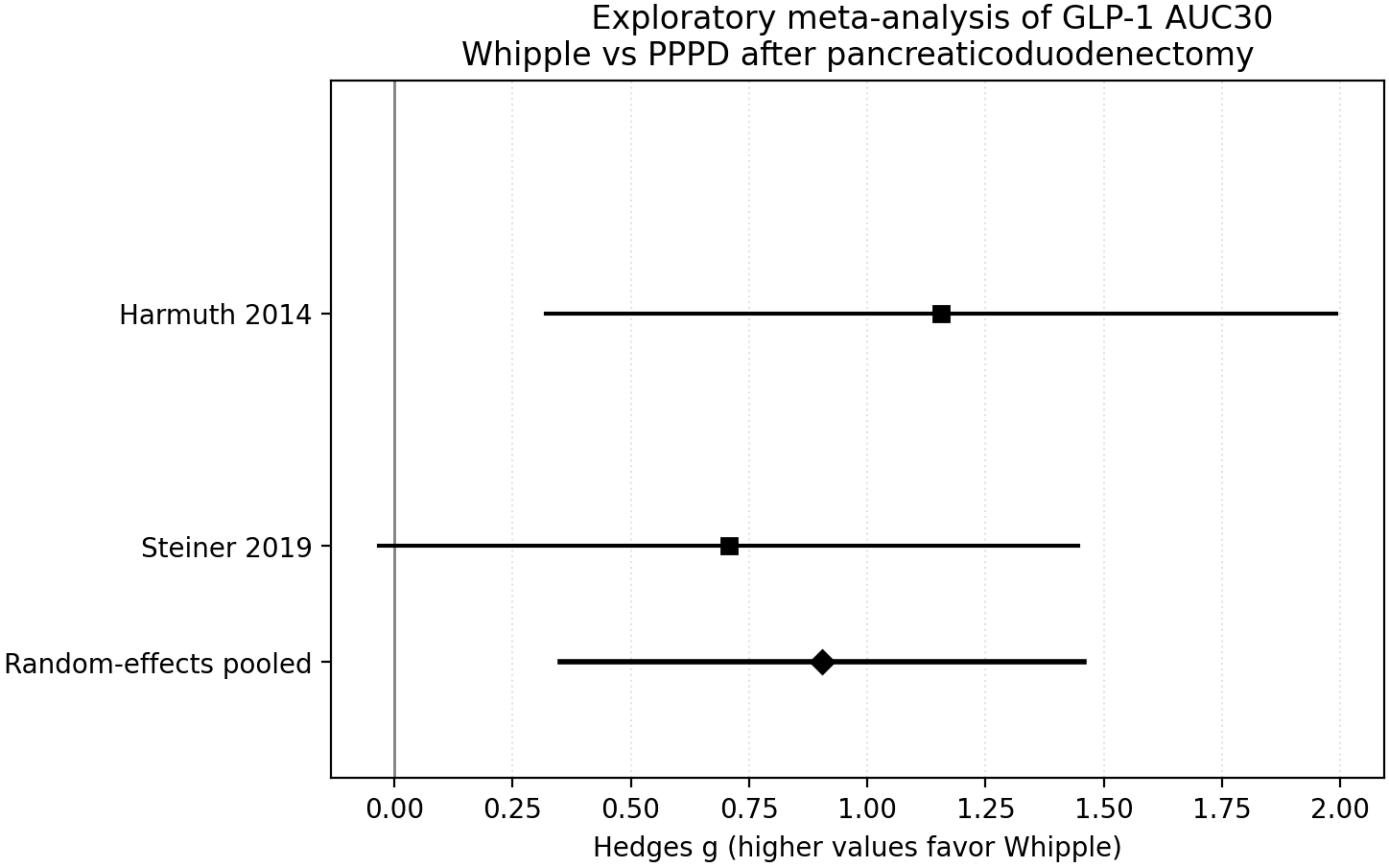
Exploratory random-effects meta-analysis of GLP-1 AUC30 after Whipple versus PPPD.

**Figure 3:**
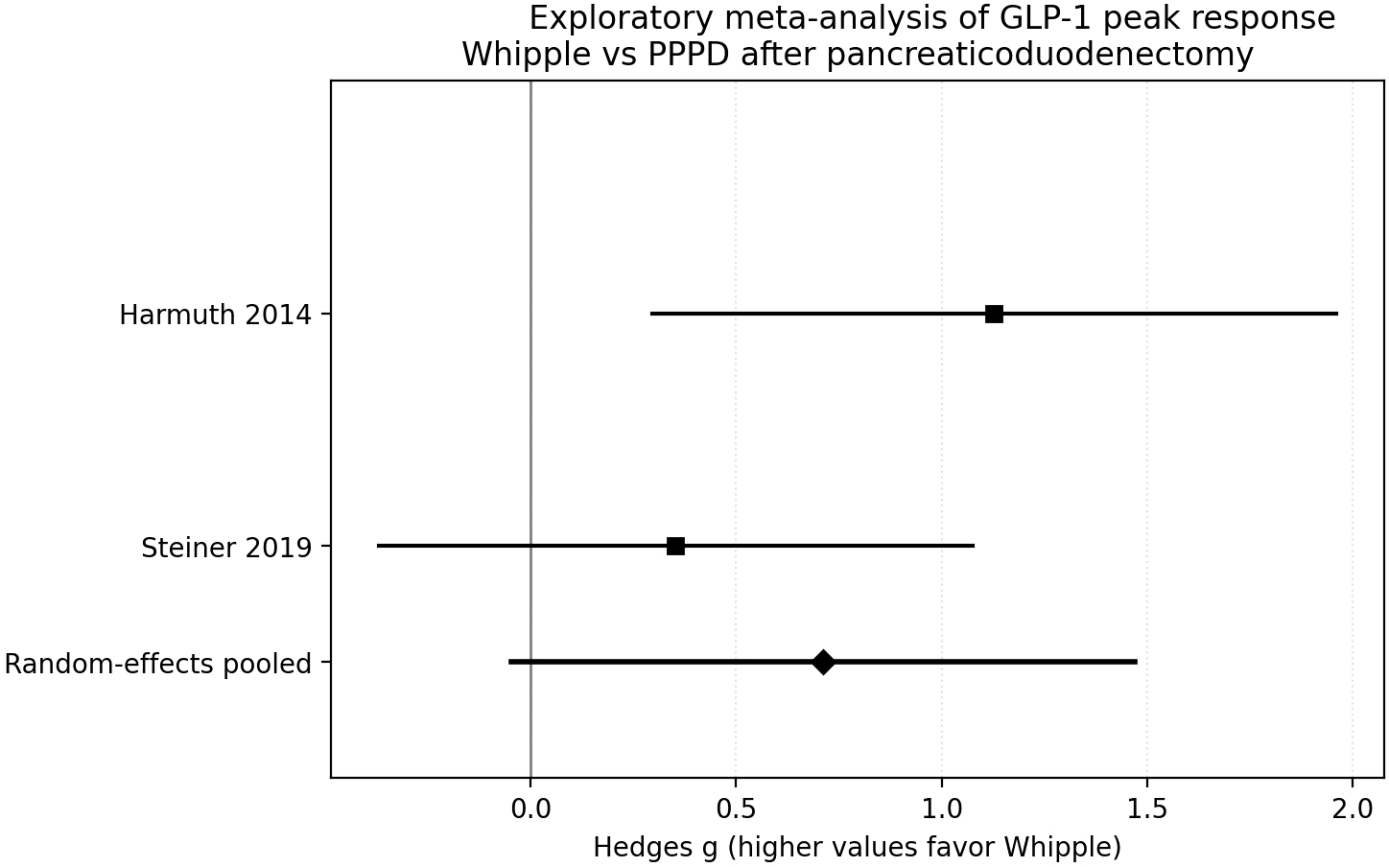
Exploratory random-effects meta-analysis of peak GLP-1 response after Whipple versus PPPD.

**Figure 4:**
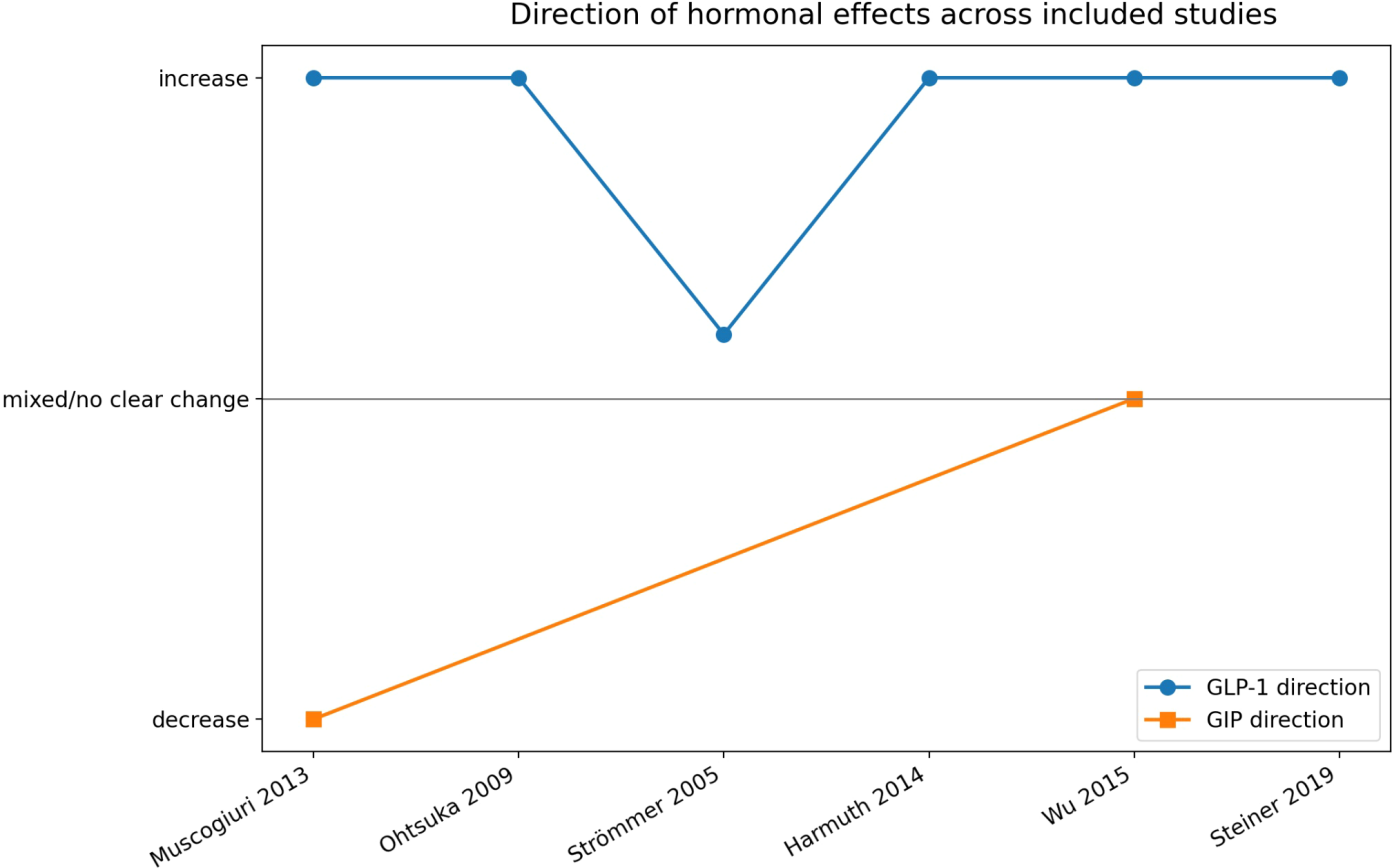
Direction of hormonal effects across included studies. Figure is intended solely as a qualitative direction-of-effect summary. The symbols indicate the reported direction of change within individual studies and should not be interpreted as pooled effect sizes, quantitative estimates, or evidence of statistical significance. Because the included studies differed in design, stimulation protocols, assay methods, postoperative timing, and outcome definitions, this figure should be viewed only as an explanatory visual summary.

Formal pooling was primarily feasible for GLP-1 outcomes reported by both Harmuth et al. and Steiner et al. as post-PD Whipple versus PPPD comparisons (7, 9, 32, 33, 41, 42). In addition, exploratory secondary pooling was performed for insulin AUC30 and glucose AUC30, as these outcomes were also reported in both studies and were considered sufficiently comparable for hypothesis-generating synthesis (7, 9, 32, 33, 41, 42). Because both studies reported medians with ranges or IQRs, approximate means and SDs were derived before random-effects pooling (7, 9, 32, 33, 41, 42). The quantitative synthesis should be regarded as strictly exploratory rather than confirmatory: each pooled comparison was based on only two studies, and therefore both the Hedges g estimates and the I² heterogeneity estimates are statistically fragile and should not be overinterpreted (7, 9, 32, 33, 41, 42). The exploratory pooled outcomes are summarized in **Table 3**. The exploratory forest plots for GLP-1 AUC30 and peak GLP-1 response are shown in **Figures 2** and **3**, respectively, and a qualitative direction-of-effect summary across the included studies is provided in **Figure 4**.

**Table 3:** Exploratory quantitative synthesis of comparable post-PD reconstruction studies.

Pooled outcome	Studies	Model	Effect size	95% CI	Heterogeneity
GLP-1 AUC30, Whipple vs PPPD	2	Exploratory random effects (Hedges g; two-study pool)	0.90	0.35 to 1.46	I² = 0%
GLP-1 peak, Whipple vs PPPD	2	Exploratory random effects (Hedges g; two-study pool)	0.71	-0.05 to 1.47	I² = 47%
Insulin AUC30, Whipple vs PPPD	2	Exploratory secondary pooling (two-study pool)	0.52	-0.08 to 1.13	I² = 21%
Glucose AUC30, Whipple vs PPPD	2	Exploratory secondary pooling (two-study pool)	-0.47	-1.00 to 0.06	I² = 0%

### 3.4 Risk of bias and certainty of evidence

All included studies were non-randomized and small (29, 30). Key recurrent concerns were selection bias, incomplete control of preoperative metabolic heterogeneity, absence of blinded laboratory assessment, and inconsistent reporting of dispersion metrics for hormonal outcomes (29, 30). The crossover feeding study minimized between-subject confounding but remained limited by very early postoperative timing and lack of randomization of feeding-route order (29, 30).

To improve transparency, the domain-based risk-of-bias judgments for each included study are summarized in **Table 4**. No study was judged to be at low risk of bias across all domains. The most frequent concerns involved confounding by baseline metabolic status, heterogeneity in surgical reconstruction and postoperative timing, incomplete reporting of hormonal assay characteristics, and limited statistical reporting due to small sample size or nonparametric summary data (29–31).

**Table 4:** Domain-based risk-of-bias summary of included studies.

Study	Selection bias	Confounding	Hormonal assay/outcome measurement	Completeness of follow-up and sampling	Statistical reporting	Overall risk-of-bias judgment
Muscogiuri 2013	Moderate	Serious	Moderate	Moderate	Serious	Serious
Ohtsuka 2009	Moderate	Serious	Moderate	Moderate	Moderate	Serious
Strömmer 2005	Moderate	Serious	Moderate	Moderate	Moderate	Serious
Harmuth 2014	Moderate	Moderate	Moderate	Moderate	Moderate	Moderate
Wu 2015	Moderate	Moderate	Moderate	Moderate	Moderate	Moderate
Steiner 2019	Moderate	Moderate	Moderate	Moderate	Moderate	Moderate

Muscogiuri et al. and Ohtsuka et al. were before-after cohorts and were therefore particularly vulnerable to confounding by postoperative timing, perioperative metabolic changes, and residual pancreatic endocrine reserve. Strömmer et al. compared patients with and without delayed gastric emptying, which introduced additional risk of confounding by postoperative clinical status. Harmuth et al. and Steiner et al. provided clinically relevant reconstruction comparisons, but their non-randomized design, small sample size, and incomplete control of baseline metabolic differences resulted in moderate overall risk of bias. Wu et al. used a within-patient feeding-route comparison, which reduced between-subject confounding; however, the very early postoperative setting and lack of randomization of feeding-route order precluded a low-risk judgment (29–31).

Overall certainty of evidence for the core conclusion, namely that GLP-1 increases after pancreaticoduodenectomy or with greater distal nutrient exposure, was judged low (30, 31). A structured GRADE-style certainty assessment is presented in **Supplementary Table 5** (30, 31). The direction of effect was coherent across several study designs; however, confidence was downgraded because of risk of bias, imprecision, indirectness related to heterogeneous stimulation protocols and postoperative timing, and partial reliance on transformed or digitized numerical data for quantitative synthesis (30, 31).

## 4 Discussion

This focused review identifies GLP-1 as the dominant incretin signal altered after pancreaticoduodenectomy (PD) (4–9). Across before–after cohorts, reconstruction comparisons, and feeding-route experiments, postprandial GLP-1 was consistently higher after PD or in post-PD configurations that enhanced distal nutrient delivery (4–9). The biological consistency of this signal is notable, given the substantial heterogeneity across included studies in postoperative timing, stimulus composition, and analytical platforms (4–9). The review also demonstrates that enhanced GLP-1 secretion should not be interpreted simplistically as universal metabolic improvement (4, 5, 8, 9, 25, 43, 44). In the earliest before–after PPPD study, GLP-1 increased while glucose tolerance worsened and insulin and C-peptide secretion declined, indicating that the endocrine consequences of pancreatic tissue loss may override the incretin rise (8, 9, 25, 43–45). Conversely, in the early postoperative PD cohort reported by Ohtsuka et al. and in the distal feeding experiment by Wu et al., glucose exposure improved under specific conditions (8, 9, 25, 43–45). These findings suggest that post-PD metabolism reflects a dynamic interplay between reduced β-cell mass, altered insulin sensitivity, gastric emptying, nutrient routing, and distal gut hormone stimulation (4, 5, 8, 9, 25, 43–45).

An important interpretative issue concerns the inconsistent distinction between active and total GLP-1 across the included studies. Active GLP-1 represents the biologically active fraction capable of receptor activation, whereas total GLP-1 also includes inactive metabolites and degradation products (2, 3). Therefore, these measures are not biologically interchangeable. Differences in assay type, sample handling, use of DPP-4 inhibitors during sample processing, and reporting of active versus total GLP-1 may influence both the magnitude and the biological interpretation of postoperative GLP-1 changes. Consequently, the recurrent increase in GLP-1 after pancreaticoduodenectomy should be interpreted as a consistent direction-of-effect signal rather than as directly comparable quantitative evidence across studies.

GIP remains the least well-characterized component of the incretin response after PD (4, 12–18, 46). In contrast to GLP-1, for which a broadly consistent direction of postoperative increase was observed across several study designs, the evidence for GIP was based on fewer studies and was substantially less consistent (4, 12–18, 46). Only two eligible studies reported quantitative GIP data (4, 12–18, 46). Muscogiuri et al. described a marked postoperative decrease after PPPD, whereas Wu et al. did not observe a clear distal-feeding effect on GIP despite a distinct GLP-1 response (4, 12–18, 46). Therefore, conclusions regarding GIP should be considered weaker and more tentative than those regarding GLP-1. This divergence is biologically plausible, as PD removes the duodenum and proximal jejunum - regions with the highest K-cell density - while simultaneously promoting more rapid distal nutrient exposure, thereby enhancing L-cell stimulation and GLP-1 release (4, 12–18, 46). This pattern is therefore better interpreted as an incretin dissociation rather than a uniform incretin enhancement and may help explain why increased GLP-1 does not consistently translate into improved postoperative glycemic control (4, 12–18, 46).

The exploratory meta-analysis should be interpreted within this mechanistic framework (7, 9, 32, 33, 41, 42). The pooled results suggest that Whipple reconstruction may be associated with greater GLP-1 responses than PPPD, particularly in the early postprandial phase (AUC30) (7, 9, 32, 33, 41, 42). However, this quantitative component was based on only two reconstruction-comparison studies; consequently, both the pooled effect-size estimates and heterogeneity estimates are highly fragile (7, 9, 32, 33, 41, 42). These findings should not be interpreted as definitive comparative-effect estimates, but rather as a structured exploratory illustration of the direction and approximate magnitude of observed differences (7, 9, 32, 33, 41, 42). The evidence base remains limited, both included studies required transformation of nonparametric summaries, and the two-study random-effects models cannot provide robust estimates of between-study heterogeneity (7, 9, 32, 33, 41, 42). Accordingly, the pooled results should be considered hypothesis-generating and reflective of a consistent mechanistic gradient rather than confirmatory meta-analytic evidence (7, 9, 32, 33, 41, 42).

### 4.1 Clinical implications

The observed pattern of enhanced postprandial GLP-1 signaling after pancreaticoduodenectomy suggests that the type of gastrointestinal reconstruction may have clinically relevant metabolic consequences (7–11, 20, 25, 38, 40, 43, 44, 47–49). Variations in nutrient routing and gastric emptying between pylorus-preserving and classic Whipple procedures could influence postoperative glycemic control, the risk of pancreatogenic diabetes, and susceptibility to dumping-like symptoms (7–11, 20, 25, 38, 40, 43, 44, 47–49). These physiological adaptations partially resemble mechanisms described after metabolic and bariatric surgery, where accelerated distal nutrient exposure augments incretin secretion and modifies enteroinsular signaling (7–11, 20, 25, 40, 43, 47, 48). From a therapeutic perspective, understanding reconstruction-dependent incretin responses may have potential implications for individualized pharmacological strategies, including the use of GLP-1 receptor agonists or other incretin-based therapies in selected post-pancreatectomy populations; however, these hypotheses require validation in prospective studies (7–11, 20, 25, 40, 43, 44, 47–50). Current clinical evidence remains insufficient to support reconstruction-driven metabolic decision-making, and future studies should integrate standardized metabolic phenotyping with detailed surgical variables (7–11, 20, 25, 38, 40, 43, 44, 47–49).

The main strengths of this review include its focused endocrine question, explicit PD-specific eligibility criteria, integration of co-reported hormonal and metabolic outcomes, and clear distinction between narrative synthesis and exploratory quantitative analysis (26–31). Limitations include reliance on the available study set and Rayyan workflow data, restriction to English-language publications, potential overlap across postoperative cohorts, and incomplete reporting of dispersion measures (26–31). A further important limitation with direct implications for interpretation is the inconsistent distinction between active and total GLP-1 across studies. Because active GLP-1 and total GLP-1 reflect biologically different analytes, differences in assay methodology, sample processing, and reporting may have contributed to between-study heterogeneity and limit the comparability of absolute GLP-1 concentrations. Therefore, the GLP-1 findings should primarily be interpreted in terms of direction and biological plausibility rather than as directly comparable quantitative estimates (2, 3, 26–31). The limited number of eligible studies reflects the specificity and clinical complexity of the research question rather than deficiencies in the search strategy (26–31).

Taken together, the available evidence suggests that pancreaticoduodenectomy induces a consistent shift toward enhanced distal gut hormone signaling, with GLP-1 as the dominant incretin mediator, although its metabolic consequences remain context-dependent (2–9, 43, 47, 48).

## 5 Conclusions

After pancreaticoduodenectomy, postprandial GLP-1 secretion generally increases, particularly when gastric emptying is faster or nutrients are delivered more distally (4–9, 43, 47, 48). Compared with GLP-1, conclusions regarding GIP remain substantially less certain because GIP outcomes were reported in fewer studies and showed less consistent patterns, suggesting either attenuation or no clear enhancement after PD (4–9, 43, 47, 48). However, these conclusions are based on only six small and heterogeneous studies including 134 participants and should therefore be interpreted with considerable caution. The available evidence remains hypothesis-generating and cannot be considered definitive or directly generalizable to all patients undergoing pancreaticoduodenectomy. Similarly, the meta-analytic component was based on only two reconstruction-comparison studies per pooled outcome; therefore, the pooled effect-size and heterogeneity estimates are statistically fragile and should be viewed as exploratory rather than confirmatory. The incretin signal alone does not determine postoperative glycemic outcome, which remains shaped by residual pancreatic endocrine reserve, reconstruction type, nutrient transit, and postoperative physiology (4–9, 43, 47, 48). Dedicated prospective PD studies should standardize mixed-meal or OGTT protocols, report paired means and SDs or raw data for GLP-1, GIP, insulin, glucagon, and glucose, and distinguish reconstruction-related effects from the consequences of pancreatic tissue loss (4–9, 43, 47, 48).

## Data Availability

The original contributions presented in the study are included in the article/**Supplementary Material**. Further inquiries can be directed to the corresponding author.
