# Supplementary material for: Changes in incretin hormone concentrations after pancreaticoduodenectomy: a systematic review and exploratory meta-analysis
Source: Front Endocrinol (Lausanne). 2026 Jun 3;17:1845925. doi: 10.3389/fendo.2026.1845925 (PMC13273729; doi:10.3389/fendo.2026.1845925)
Supplement: Supplementary file 1 [file Table1.docx]

**PRISMA 2020 Checklist**

*Manuscript: Changes in incretin hormone concentrations after pancreaticoduodenectomy: a systematic review and exploratory meta-analysis*

**PROSPERO: 1345848**

| **Item** | **Checklist requirement** | **Location in manuscript / supplement** |
| --- | --- | --- |
| **Title** | Identify the report as a systematic review, meta-analysis, or both. | Title page |
| **Abstract** | Structured abstract summarizing background, methods, results, limitations, and conclusions. | Abstract |
| **Rationale and objectives** | Describe the rationale and explicit objectives/questions. | Introduction |
| **Eligibility criteria** | Specify inclusion and exclusion criteria. | Methods – Eligibility criteria |
| **Information sources and search strategy** | Specify all databases, date of last search, and full strategies. | Methods; Supplementary Table S2 |
| **Selection process** | Describe screening methods and software. | Methods |
| **Data collection process** | Describe extraction procedures and digitization/transformation rules. | Methods |
| **Risk of bias** | Describe tool and approach used to assess bias. | Methods; Supplementary Table S4 |
| **Synthesis methods** | Describe narrative synthesis and exploratory random-effects meta-analysis. | Methods |
| **Study selection results** | Present numbers screened, assessed, and included, ideally with flow diagram. | Figure 1; Results |
| **Study characteristics** | Provide design, population, stimulus, outcomes, and timing. | Table 1; Supplementary Table S3 |
| **Results of syntheses** | Summarize all included findings and pooled estimates where applicable. | Results; Tables 2–3; Figures 2–4 |
| **Discussion** | Discuss interpretation, limitations, and implications. | Discussion |
| **Registration and support** | PROSPERO registration number: 1345848. Funding statement, ethics statement, data availability statement, and generative AI statement are included in the manuscript. | Abstract; Methods; Funding; Ethics statement; Data availability statement; Generative AI statement |
