# Supplementary material for: Changes in incretin hormone concentrations after pancreaticoduodenectomy: a systematic review and exploratory meta-analysis
Source: Front Endocrinol (Lausanne). 2026 Jun 3;17:1845925. doi: 10.3389/fendo.2026.1845925 (PMC13273729; doi:10.3389/fendo.2026.1845925)
Supplement: Supplementary file 2 [file Table2.docx]

Supplementary Material
Changes in incretin hormone concentrations after pancreaticoduodenectomy

Prepared for Frontiers in Endocrinology. PROSPERO registration number: 1345848. The PRISMA 2020 checklist is provided as a separate submission file.

# Supplementary Table S2. Reconstructed search strategies

The review workflow indicates searches of PubMed, Embase, and Scopus up to 27 March 2026. The strings below were formatted from the documented workflow for submission-ready reporting. A full PubMed search strategy is reported in Table S2 for submission-ready transparency.

| Database | Search strategy reported from workflow files |
| --- | --- |
| PubMed | ("Pancreaticoduodenectomy"[Mesh] OR pancreatoduodenectomy OR pancreaticoduodenectomy OR "Whipple procedure" OR Whipple OR PPPD OR "pylorus preserving pancreatoduodenectomy") AND ("Incretins"[Mesh] OR incretin* OR "GLP-1" OR "glucagon-like peptide-1" OR GIP OR "glucose-dependent insulinotropic polypeptide" OR "gut hormone*" OR enteroendocrine OR "GLP-2" OR "peptide YY" OR PYY OR "enteroinsular axis") |
| Embase | ('pancreaticoduodenectomy'/exp OR pancreatoduodenectom*:ti,ab,kw OR pancreaticoduodenectom*:ti,ab,kw OR 'whipple procedure':ti,ab,kw OR whipple:ti,ab,kw OR pppd:ti,ab,kw OR 'pylorus preserving pancreatoduodenectomy':ti,ab,kw) AND ('incretin'/exp OR incretin*:ti,ab,kw OR 'glucagon like peptide 1'/exp OR 'glp 1':ti,ab,kw OR gip:ti,ab,kw OR 'glucose dependent insulinotropic polypeptide':ti,ab,kw OR 'gut hormone*':ti,ab,kw OR enteroendocrine:ti,ab,kw OR 'glp 2':ti,ab,kw OR 'peptide yy':ti,ab,kw OR pyy:ti,ab,kw OR 'enteroinsular axis':ti,ab,kw) |
| Scopus | TITLE-ABS-KEY ( pancreatoduodenectomy OR pancreaticoduodenectomy OR "Whipple procedure" OR Whipple OR PPPD OR "pylorus preserving pancreatoduodenectomy" ) AND TITLE-ABS-KEY ( incretin* OR "GLP-1" OR "glucagon-like peptide-1" OR GIP OR "glucose-dependent insulinotropic polypeptide" OR "gut hormone*" OR enteroendocrine OR "peptide YY" OR PYY OR "enteroinsular axis" ) |

# Supplementary Table S3. Detailed extracted study-level findings

| **Study** | **Participants / design** | **Stimulus and timing** | **Principal hormonal findings** | **Additional metabolic findings** |
| --- | --- | --- | --- | --- |
| Muscogiuri 2013 | 10 overweight, non-diabetic adults; prospective before-after PPPD | 830-kcal mixed meal; assessed before and after recovery (~30-50 days) | GLP-1 increased; GIP decreased; glucagon increased after PPPD | Insulin and C-peptide decreased; fasting and postprandial glucose increased |
| Ohtsuka 2009 | 17 patients undergoing mixed PD types; prospective before-after | 75-g OGTT before and 1 month after PD | Postoperative GLP-1 concentration significantly higher than preoperative value | Glucose and insulin concentrations lower postoperatively; HOMA-IR improved |
| Strömmer 2005 | 31 patients after PD; 9 DGE and 22 non-DGE | Liquid meal + paracetamol on postoperative day 11 | Total and incremental GLP-1 responses similar in DGE and non-DGE groups | PYY and neurotensin reduced with DGE; motilin similar |
| Harmuth 2014 | 26 tumor-free subjects after PD; Whipple vs PPPD (13/13) | 75-g OGTT + paracetamol; median 23 months after PD | Whipple showed higher GLP-1 peak, AUC30, and AUC180 than PPPD | Faster gastric emptying after Whipple; insulin sensitivity favored Whipple |
| Wu 2015 | 20 patients after PD; within-patient proximal vs distal feeding | Enteral meal test on postoperative day 5 | Distal feeding increased GLP-1 AUC0-120; no clear GIP enhancement | Insulin and C-peptide AUC increased; glucose AUC decreased with distal feeding |
| Steiner 2019 | 30 tumor-free post-PD subjects; Whipple vs PPPD (15/15) | Mixed meal + paracetamol; median 12 months after PD | Whipple and faster emptying associated with higher GLP-1 peak and AUC30 | Lower HbA1c and better insulin sensitivity associated with greater GLP-1 release |

# Supplementary Table S4. Risk-of-bias assessment (design-specific pragmatic evaluation)

| **Study** | **Selection** | **Outcome measurement** | **Confounding / comparability** | **Completeness** | **Overall** |
| --- | --- | --- | --- | --- | --- |
| Muscogiuri 2013 | Moderate concern | Low concern for assay; moderate for timing variability | Moderate concern | Moderate concern | Moderate |
| Ohtsuka 2009 | Moderate concern | Moderate concern (limited assay detail) | Moderate concern | Moderate concern | Moderate |
| Strömmer 2005 | Moderate concern | Low concern | Moderate concern | Moderate concern | Moderate |
| Harmuth 2014 | Moderate concern | Low concern | Moderate concern | Low concern | Moderate |
| Wu 2015 | Moderate concern | Moderate concern | Moderate concern | Low concern | Moderate |
| Steiner 2019 | Moderate concern | Low concern | Moderate concern | Low concern | Moderate |

No included study was judged low risk overall because all were small, non-randomized clinical studies.

# Supplementary Table S5. Certainty of evidence (GRADE-style narrative summary)

| **Question** | **Body of evidence** | **Certainty** | **Reasons for downgrading** |
| --- | --- | --- | --- |
| Does GLP-1 increase after PD or with greater distal nutrient exposure? | 6 small observational or non-randomized studies; direction broadly consistent | Low | Risk of bias, imprecision, indirectness across stimuli and postoperative timing |
| Does GIP increase after PD? | 2 studies with inconsistent findings | Very low | Risk of bias, inconsistency, imprecision |
| Is Whipple associated with higher GLP-1 than PPPD after PD? | 2 observational comparative cohorts with transformable summary statistics | Low | Risk of bias, imprecision, transformed nonparametric summaries |
| Do distal nutrient delivery strategies improve glycemic exposure after PD? | 1 crossover experiment and supportive mechanistic comparisons | Low | Single-study dependence, early postoperative setting, indirectness |

# Supplementary Table S6. Native Rayyan record-level table of excluded full-text studies

The table below provides the 1:1 record-level list of all excluded full-text studies (n = 30). Final study status and exclusion reasons were derived directly from the native Rayyan customization-log export rather than reconstructed from screenshots. The same native log confirmed six included full-text records, yielding 36 full texts assessed in total.

| **Rayyan article ID** | **Excluded full-text study** | **Native Rayyan exclusion reason** |
| --- | --- | --- |
| 462923053 | Different incretin responses after pancreatoduodenectomy and distal pancreatectomy. | wrong outcome |
| 462923063 | Quality of life, nutritional status, and gastrointestinal hormone profile following the Whipple procedure. | wrong outcome |
| 462923066 | Rikkunshito increases appetite by enhancing gastrointestinal and incretin hormone levels in patients who underwent pylorus-preserving pancreaticoduodenectomy: A retrospective study. | wrong population |
| 462923068 | Reduced incretin effect precedes diabetes development following duodenopancreatectomy in individuals without diabetes. | wrong publication type |
| 462923071 | Insulin resistance alters islet morphology in nondiabetic humans. | wrong population |
| 462923072 | Gastric inhibitory polypeptide secretion after radical pancreatoduodenectomy. | wrong outcome; wrong population |
| 462923074 | Importance of Intestinal Environment and Cellular Plasticity of Islets in the Development of Postpancreatectomy Diabetes. | wrong outcome |
| 462923076 | Gastric inhibitory polypeptide (GIP), gastrin and insulin: response to test meal in coeliac disease and after duodeno-pancreatectomy. | wrong publication type |
| 462923077 | Gastric acid secretion and gut hormone release in patients undergoing pancreatico-duodenectomy. | wrong outcome |
| 462923084 | The enteral insulin-stimulation after Whipple's operation. | wrong outcome |
| 462923085 | [Gut hormone profiles after various types of gastrointestinal surgery]. | wrong population |
| 462923087 | [Changes of responses of gastrointestinal hormones after pancreatectomies]. | wrong outcome |
| 462923158 | Reduced Incretin Effect Predicts Diabetes Appearance in a Cohort of Nondiabetic Subjects after Acute Beta-Cell Mass Reduction. | wrong study design |
| 462923168 | Reversibility of secretion of gastric inhibitory polypeptide in long-term survivors after pancreatoduodenectomy. | wrong outcome |
| 462923179 | Reduced incretin effect is an early sign of diabetes appearance. A study in a human model of beta cell mass reduction. | wrong population |
| 462923186 | Release of gastric inhibitory polypeptide (GIP) and gastrin after a test meal with a low glucose load in patients after B II resection, proximal duodenopancreatectomy (PDP) and jejunoileal bypass. | wrong population |
| 462923200 | Differential changes in glucagon and GLP-1 after partial pancreatoduodenectomy might represent an attempt to increase the incretin effect in insulin-resistant subjects. | wrong outcome |
| 462923203 | The change of glucose metabolism with GLP-1 after pancreatectomy. | wrong population |
| 462923209 | The impact of body position on gastric emptying after pancreaticoduodenectomy. | wrong outcome |
| 462923213 | In insulin-resistant subjects, islet functional changes might represent an attempt to increase the incretin effect. | wrong population |
| 462923219 | Impaired beta cell function and reduced incretin effect following surgical acute beta cell loss in humans. | wrong study design |
| 462923220 | Surgical acute beta-cell loss determines beta-cell dysfunction and reduced incretin effect in humans. | wrong study design |
| 462923221 | Gastric emptying and mode of operation independently enhance the release of GLP-1 after a mixed meal and improve glycemic control in subjects having undergone Whipple's procedure and pylorus preserving pancreaticoduodenectomy (PPPD). | wrong population |
| 462923228 | Early dumping and reactive hypoglycemia after pancreaticoduodenectomy: The relationship between gastric emptying, pylorus preservation and glycemic control. | wrong outcome |
| 462923233 | In vivo beta cell glucose sensitivity regulates ex vivo islet size, transdifferentiation and GLP1 immunoreactivity in the alpha cells in humans. | wrong population |
| 462923241 | Alterations in islet morphology as a function of insulin sensitivity in humans. | wrong study duration |
| 462923244 | Gastric emptying, ensuing GLP-1 release and insulin sensitivity after partial pancreaticoduodenectomy: Improved glycemic control in cases without pylorus preservation (Whipple procedure). | wrong study design |
| 462923245 | Accelerated gastric emptying after Whipple procedure wards off diabetes mellitus through rapid gastric emptying and high postprandial concentrations of glucagon-like peptide-1 (GLP-1). | wrong publication type |
| 462923249 | Gastric emptying, if accelerated after pancreaticoduodenectomy induces an exaggerated release of GLP-1 and is associated with lower fasting glucose and HbA1c levels in patients under age 70: A comparison between Whipple's operation and pylorus preservation. | wrong publication type |
| 462923252 | Effects of Pancreaticoduodenectomy on Glucose Metabolism. | wrong outcome |

Data availability and ethics note: All extracted data used in the review are contained in the manuscript and this Supplementary Material. Ethical review was not required because the review analyzed previously published studies only.
